# Management of pain in Fabry disease in the UK clinical setting: consensus findings from an expert Delphi panel

**DOI:** 10.1186/s13023-023-02796-1

**Published:** 2023-07-21

**Authors:** Karolina M. Stepien, Alexander Broomfield, Duncan Cole, Patrick B. Deegan, Stuart Forshaw-Hulme, Derralynn Hughes, Ana Jovanovic, Liz Morris, Alison Muir, Uma Ramaswami

**Affiliations:** 1 The Mark Holland Metabolic Unit, Salford Royal NHS Foundation Trust, Salford, UK; 2 Willink Biochemical Genetics Unit, Manchester Centre for Genomic Medicine, St Mary’s Hospital, Manchester University NHS Foundation Trust, Manchester, UK; 3 Department of Medical Biochemistry and Immunology, University Hospital of Wales, Cardiff, Wales UK; 4 Lysosomal Disorders Unit, Addenbrooke’s Hospital, Cambridge, UK; 5 Lysosomal Storage Disorders Unit, Royal Free London NHS Foundation Trust, London, UK; 6 Belfast Heart Centre, Royal Victoria Hospital, Belfast, UK

**Keywords:** Fabry disease, Pain, Delphi panel, Neuropathic pain, Lyso-GB3, Analgesic therapy

## Abstract

**Background:**

Fabry disease is a rare, X-linked inherited lysosomal storage disorder, that manifests as a heterogeneous disease with renal, cardiac and nervous system involvement. The most common pain experienced by people with Fabry disease are episodes of neuropathic pain reported in up to 80% of classical hemizygous male patients and up to 65% of heterozygous female patients. No clear consensus exists within UK clinical practice for the assessment and management of pain in Fabry disease based on agreed clinical practice and clinical experience. Here we describe a modified Delphi initiative to establish expert consensus on management of pain in Fabry disease in the UK clinical setting.

**Methods:**

Delphi panel members were identified based on their demonstrated expertise in managing adult or paediatric patients with Fabry disease in the UK and recruited by an independent third-party administrator. Ten expert panellists agreed to participate in two survey rounds, during which they remained anonymous to each other. Circulation of the questionnaires, and collection and processing of the panel’s responses were conducted between September 2021 and December 2021. All questions required an answer.

**Results:**

The Delphi panel reached a consensus on 21 out of 41 aspects of pain assessment and management of pain in Fabry disease. These encompassed steps in the care pathway from the goals of therapy through to holistic support, including the use of gabapentin and carbamazepine as first-line analgesic medications for the treatment of neuropathic pain in Fabry disease, as well as the proactive management of symptoms of anxiety and/or depression associated with Fabry pain.

**Conclusions:**

The consensus panel outcomes reported here have highlighted strengths in current UK clinical practice, along with unmet needs for further research and agreement. This consensus is intended to prompt the next steps towards developing clinical guidelines.

## Introduction

Fabry disease is a rare, progressive X-linked inherited lysosomal storage disorder that manifests as a multisystem disease. Fabry disease is caused by mutations in the *GLA* gene that encodes the lysosomal enzyme α-galactosidase A (α-gal A) [1], with consequent pathological accumulation of glycosphingolipids [2], including globotriaosylceramide (Gb3) and its deacetylated derivative globotriaosylsphingosine (lyso-GB3), predominantly in vascular endothelium, smooth muscle cells and the peripheral nervous system. This results in a heterogeneous disease with renal, cardiac and nervous system involvement. The phenotypic spectrum of Fabry disease includes the classical severe phenotype, wherein the worst affected cases symptoms can manifest in early childhood [3] and significantly reduce both the quality of life and capacity for normal social integration with wide ranging impact on academic attendance and performance of children and young adults.

Pain is an early symptom reported by many patients with the classic phenotype of Fabry disease, with prevalence of 60–70% in male patients and 40–60% of female patients [4–7]. Different types of pain are evident in Fabry disease, including chronic episodes of burning, stabbing or shooting pain, with intensity ranging from low to severe [8]. Also, episodes of severe, debilitating acute pain in hands and feet, with radiating acroparaesthesia that can persist despite the elimination of the initial pain trigger, are a feature of Fabry disease and are known as ‘Fabry crises’, which can last for minutes or continue for weeks [4, 8]. Fabry crises may be triggered by factors such as changes in environmental or body temperature, stress, exercise or exertion, large meal portions (in cases of gastrointestinal pain) as well as concurrent illness [8, 9].

The most common pain experienced by people with Fabry disease is acroparaesthesia. This presents as episodes of neuropathic pain, and is reported in up to 80% of classical hemizygous male patients and up to 65% of heterozygous female patients [10–12]. The pathophysiology of pain in Fabry disease is poorly understood, but it is known to be associated with residual α-gal A activity, being more severe in classical phenotypes with no residual enzymatic activity [13, 14]. However, individuals with the same mutation in the *GLA* gene can experience considerable variation in pain symptoms and severity, confirming the unpredictability of pain in Fabry disease [4]. The pathophysiology of pain in Fabry disease involves autonomic and peripheral nervous systems [15], and is linked to small and large fibre neuropathy [16–18], including myelinated Aδ and unmyelinated C-fibres [16, 19], as well as ion-channel abnormalities [14, 20, 21]. Evidence supports a direct effect of lyso-Gb3 accumulation in dorsal root ganglia of sensory neurons, with subsequent pain production [14]. Small-fibre neuropathy may lead to painful hyposensitivity to warm or cold temperatures, as well as hypersensitivity to mechanical pain. Because of this, the presence of Fabry-specific small-fibre neuropathy should be a component of a confirmed diagnosis and initiation of therapeutic interventions [22]. Abdominal pain has been associated with impaired autonomic function [23].

Recommendations for the assessment and treatment of pain in Fabry disease are emerging [22, 24, 25], but no clear consensus exists within UK clinical practice for the assessment and management of pain in Fabry disease, that is based on clinical practice and clinical experience. Here we describe a modified Delphi initiative to establish an expert consensus on management of pain in patients with Fabry disease in the UK clinical setting.

## Methods

**Fig. 1:**
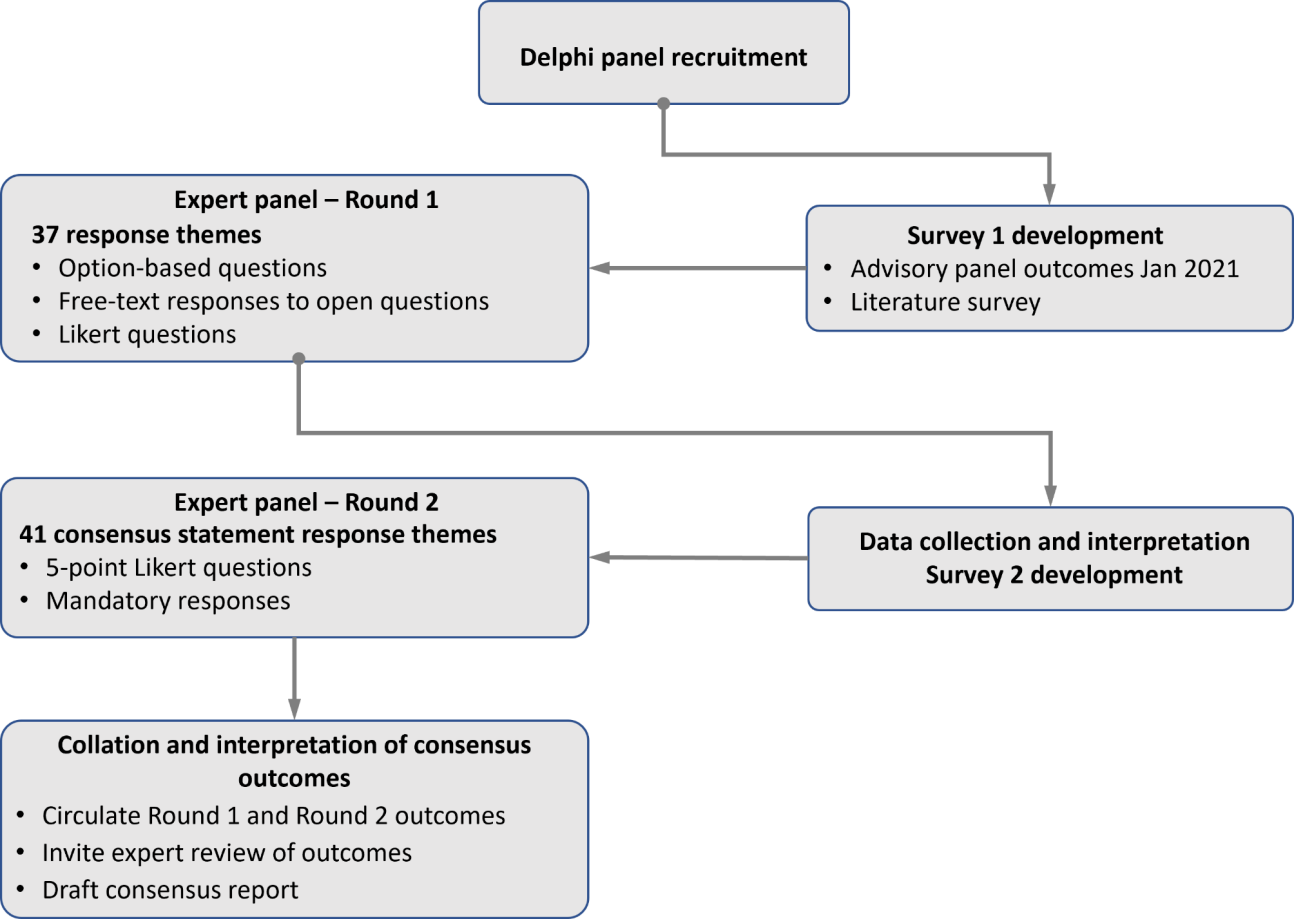
Modified Delphi methodology for consensus on pain in Fabry disease. (All stages of the Delphi process were managed by an independent third-party administrator. Expert panel responses were gathered anonymously via an online survey platform. During the two survey rounds, only the administrator knew the identities of responding panellists, but no identifying information was shared between the panel members. No feedback on the outcomes of the Round 1 survey were provided prior to Round 2. The anonymity of the responses provided by the Delphi panel members was maintained throughout the process, including after distribution of the survey outcomes)

The Delphi process is a widely used [26], validated technique for developing an expert consensus on clinical needs and approaches, and has previously been used for clinical assessment and management of patients with Fabry disease [27–30]. The serial use of questionnaires and the maintenance of anonymity minimises the risk that an expert group may conform to a dominant opinion. The modified Delphi process used is described below and summarised in Fig. 1.

### Selection of expert panel

Panel members were identified based on their demonstrated expertise in managing adult or paediatric patients with Fabry disease in the UK, their acknowledged participation in national or regional Fabry disease management programmes, involvement in clinical trials or clinical studies centred on outcomes in Fabry disease, or authorship of relevant peer-reviewed publications. Panellists were recruited by an independent third-party administrator (Bite Medical Consulting, Cambridge, UK). Thirteen panellists were invited, 10 of whom agreed to participate. One expert declined to participate due to time commitments, one declined because of a perceived conflict of interest, and one did not respond during the recruitment period.

### The Delphi process

The independent third-party administrator drafted a study protocol that developed key themes expressed by paediatric Fabry disease experts during a UK advisory board meeting held in January 2021. A non-exhaustive literature search was also conducted by the administrator to inform aspects of the initiative, from which 128 publications were identified. All stages of the initiative were managed by the independent third-party administrator. Panel responses were gathered anonymously via an online survey platform (SurveyMonkey.com). During the two survey rounds, only the administrator knew the identities of responding panellists, but no identifying information was shared between the panel members. Panellists remained anonymous to each other throughout the Delphi stages.

Circulation of the questionnaires, and collection and processing of the panel’s responses were conducted between September 2021 and December 2021. All questions required an answer. No controlled feedback was provided to panellists during or between survey rounds. Given the focus and scope of the treatment area under consideration, two rounds of survey activity were planned. In round 1, information was collected regarding the panellist’s individual clinical practices, experience and attitudes. Round one was a mix of option-based choices, free-text responses to open questions and attitudes-based 5-point Likert scale (1 = Strongly disagree; 2 = Disagree; 3 = Neither Agree nor Disagree; 4 = Agree; 5 = Strongly Agree). Round 1 comprised 37 individual questions.

Based on the first-round survey responses, the second-round survey was compiled, comprising 17 survey question elements, with 41 consensus statements that required panellists to rate the importance of each statement on a 5-point Likert scale as above. Agreement on consensus items would be reached if a consensus score of ≥ 4 was awarded by > 67% of the panel.

### Statistical analyses

The Delphi panel was not undertaken for research purposes and no hypotheses were tested. Basic statistical analyses were performed only to collect and collate responses.

## Results

### Goals of pain therapy in Fabry disease

The impact of pain in Fabry disease is significant, both for the patient and for their families. Concern and anxiety commonly accompany episodes of Fabry pain. Adults may report workplace absences and children may experience absences from school, with a frequency associated with the extent of pain, and will also have restricted involvement in non-academic activities [31, 32]. Reduced academic performance has also been reported for children with Fabry disease [33]. However, only 30% of the expert panel indicated this as a notable consequence, which did not include the paediatric specialist panellists, although other chronic conditions with associated pain, such as neurofibromatosis or headache, have reported learning difficulties in children [34, 35]. Throughout life, painful episodes of Fabry disease can render sufferers unable to work or study, with associated loss of income and difficulty in sustaining relationships. These impacts can lead to mental health consequences, with depression, social withdrawal and poor quality of life.

In this context, consensus was reached for 4 goals of pain therapy in Fabry disease (Table 1). These were:

**Table 1:** Consensus criteria for assessing and managing pain in Fabry disease

Consensus statement			Mean score (out of 5)	Level of consensus
Goals of therapy for neuropathic pain				
1	To reduce or maintain pain at levels that have a minimal impact on activities of daily living		4.7	100%
2	To reduce or maintain pain at levels that have a minimal impact on educational attendance and performance		4.6	90%
3	To reduce or maintain pain at levels that minimise the impact on day-to-day family life		4.4	80%
4	To minimise psychological stress associated with neuropathic pain		4.5	90%
**Assessment of neuropathic pain in Fabry disease**				
6	A thorough physical examination is an important part of assessing and diagnosing neuropathic pain in Fabry disease		3.8	70%
7	The Brief Pain Inventory (BPI) is an effective tool for documenting and monitoring neuropathic pain in Fabry disease		4	90%
8	Patients with neuropathic pain should be provided with pain diaries or apps and instructed in how to use them		4	80%
**Stressors and triggers of neuropathic pain in Fabry disease**				
13	Avoidance of stressors/triggers of neuropathic pain should be advised in all patients with Fabry disease		4.2	90%
14	Support for avoidance/triggers of neuropathic pain should be provided (e.g., clinical letters to schools or workplaces)		4.5	100%
**First-line analgesic medication of neuropathic pain in Fabry disease**				
16	Gabapentin can be used as a first-line analgesic medication for the treatment of neuropathic pain in Fabry disease		3.9	70%
17	Carbamazepine can be used as a first-line analgesic medication for the treatment of neuropathic pain in Fabry disease		4.4	90%
18	If the initial treatment is not effective or is not tolerated, switching to another first-line analgesic medication can be considered		4.4	90%
**Second-line analgesic medication of neuropathic pain in Fabry disease**				
No consensus reached				
**Treatment responses to neuropathic pain medications in Fabry disease**				
29		Treatment responses to neuropathic pain medication should be actively monitored using pain diaries and/or pain apps	4.2	90%
30		Treatment responses to neuropathic pain medication should be actively monitored using repeated application of pain-assessment questionnaires	4.3	90%
31		For neuropathic pain that is refractory to first- and second-line medications, expert pain prescribers should be consulted	4.9	100%
**Treatment of Gastrointestinal (GI) pain in Fabry disease**				
32		Metoclopramide can be used to treat symptoms of nausea/vomiting	3.6	80%
33		Loperamide can be used to treat symptoms of diarrhoea	3.9	90%
**Assessment and treatment of anxiety and depression in Fabry disease pain**				
37		Patients with Fabry disease pain should be assessed for associated symptoms of anxiety/depression using a validated scoring tool (e.g., CES-D)	4.2	100%
38		Selection of analgesic medication for neuropathic pain in Fabry disease should accommodate symptoms of anxiety and/or depression	3.9	90%
39		Cognitive behavioural therapy (CBT) should be considered for patients with Fabry disease pain AND anxiety and/or depression	3.7	80%
40		Psychological services should be included in the MDT in cases of Fabry disease pain.	4.7	100%

There were 41 consensus statements in the second-round survey. Consensus statements were scored for agreement against a 5-point Likert scale (1 = strongly disagree; 2 = disagree; 3 = neither agree nor disagree; 4 = agree; 5 = strongly agree). Statements awarded an agreement score ≥ 4 by > 67% of panellists achieved consensus. In the table, mean Likert scores are provided along with the percentage consensus score. Each consensus statement is numbered from 1 to 41, thus the numbers in the table reflect only those statements that achieved consensus

1. To reduce or maintain pain at levels that have a minimal impact on activities of daily living.
2. To reduce or maintain pain at levels that have a minimal impact on educational attendance and performance.
3. To reduce or maintain pain at levels that minimise the impact on day-to-day family life.
4. To minimise psychological stress associated with neuropathic pain.

In achieving consensus on these goals, it was acknowledged that measuring the impact on educational performance and family life may be difficult.

### Assessment of neuropathic pain in Fabry disease

In the first-round survey across the Delphi panel, a wide selection of attitudes and practices were reported in the context of assessment of patients experiencing pain in Fabry disease. This included the use of different pain-assessment tools, most predominantly the Brief Pain Inventory (BPI), the FOS-Mainz Severity Score Index and the EQ-5D. The paediatric specialists on the panel also used the Fabry-specific health and pain questionnaire (FPHPQ). All of the Delphi panellists reported that access to specialist pain services was part of the assessment process, if required. Ultimately, only 3 of 7 possible recommendations achieved a consensus across the Delphi panel (Table 1). These were:

1. A thorough physical examination is an important part of assessing and diagnosing neuropathic pain in Fabry disease.
2. The Brief Pain Inventory (BPI) is an effective tool for documenting and monitoring neuropathic pain in Fabry disease.
3. Patients with neuropathic pain should be provided with pain diaries or apps and instructed on how to use them.

In the context of the needs for effective assessment of pain in Fabry disease, a thorough physical examination was accepted as a requirement (Table 1), but a detailed family history was not seen as diagnostic for neuropathic pain in Fabry disease (Table 2), although it can be helpful in confirming aspects of pain aetiology. It was not felt that all patients presenting with signs or symptoms of neuropathic pain should undergo assessment by a specialist pain service. However, a pain specialist should be included as part of the Fabry multi-disciplinary team (MDT) in case of the need for a consultation. Amongst assessment tools, only the BPI achieved consensus for use as part of pain assessment in Fabry disease, with some support for application of the FOS-Mainz Severity Score Index or the EQ-5D. The BPI measures pain intensity and the effect of pain on daily activity and quality of life, identifies the location of pain and assesses the effectiveness of recent pain relief medication [36]. The BPI is a validated tool and has been used in clinical practice since 1994 across various disease areas, including oncology, musculoskeletal disease and post-surgical pain [36]. It can be completed by patients in a short time and is emphasised, as well as the EQ-5D, for use in Fabry disease registries [37].

**Table 2:** Consensus not achieved for assessing and managing pain in Fabry disease

Consensus statement		Mean score (out of 5)	Level of consensus
Goals of therapy for neuropathic pain			
5	To reduce or maintain their pain at levels that reduce the need for further pain medication	3.5	50%
**Assessment of neuropathic pain in Fabry disease**			
9	All patients presenting with signs or symptoms of neuropathic pain should undergo assessment by a specialist pain service	3	30%
10	A detailed family history is an important part of assessing and diagnosing neuropathic pain in Fabry disease	3.4	60%
11	The FOS-Mainz Severity Score Index is an effective tool for documenting and monitoring neuropathic pain in Fabry disease	3.4	40%
12	The EQ-5D is an effective tool for documenting and monitoring neuropathic pain in Fabry disease	3.6	50%
**Stressors and triggers of neuropathic pain in Fabry disease**			
15	Avoidance of stressors/triggers can be an effective pain-management strategy for many patients without an initial need for analgesic medication	3.4	40%
**First-line analgesic medication of neuropathic pain in Fabry disease**			
19	Pregabalin can be used as a first-line analgesic medication for the treatment of neuropathic pain in Fabry disease	3.6	50%
20	Amitriptyline can be used as a first-line analgesic medication for the treatment of neuropathic pain in Fabry disease	3.2	50%
21	Nortriptyline can be used as a first-line analgesic medication for the treatment of neuropathic pain in Fabry disease	2.7	10%
22	Duloxetine can be used as a first-line analgesic medication for the treatment of neuropathic pain in Fabry disease	2.9	30%
23	Venlafaxine can be used as a first-line analgesic medication for the treatment of neuropathic pain in Fabry disease	2.6	0%
**Second-line analgesic medication of neuropathic pain in Fabry disease**			
24	Topical capsaicin can be used as a second-line analgesic medication for the treatment of neuropathic pain in Fabry disease	3.4	40%
25	Lidocaine patches can be used as a second-line analgesic medication for the treatment of neuropathic pain in Fabry disease	3.5	50%
26	Tramadol can be used as a second-line analgesic medication for the treatment of neuropathic pain in Fabry disease	3.2	30%
27	Codeine can be used as a second-line analgesic medication for the treatment of neuropathic pain in Fabry disease	3.6	50%
28	Oxycodone can be used as a second-line analgesic medication for the treatment of neuropathic pain in Fabry disease	3.3	40%
**Treatment of Gastrointestinal (GI) pain in Fabry disease**			
34	GI symptoms & abdominal pain can be managed using dietetic advice about meal constituents, portion sizes, meal timings and frequency	3.4	60%
35	Domperidone can be used to treat symptoms of nausea/vomiting	3.6	60%
36	Domperidone can be used to treat painful GI symptoms	3.5	50%
**Assessment and treatment of anxiety and depression in Fabry disease pain**			
41	Cognitive behavioural therapy (CBT) should be considered for ALL patients with Fabry disease pain	2.7	30%

There were 41 consensus statements in the second-round survey. Consensus statements were scored for agreement against a 5-point Likert scale (1 = strongly disagree; 2 = disagree; 3 = neither agree nor disagree; 4 = agree; 5 = strongly agree). Statements awarded an agreement score ≥ 4 by > 67% of panellists achieved consensus. In the table, mean Likert scores are provided along with the percentage consensus score. Each consensus statement is numbered from 1 to 41, thus the numbers in the table reflect only those statements that achieved consensus

The importance of pain diaries and apps, such as the Fabry Pain Diary and the Fabry Pain App, was highlighted and there was a strong consensus (80%) that patients with Fabry disease should be trained to use them, in order to gauge how their pain may be affecting daily activities. Other non-Fabry chronic pain apps are available, with some evidence that they can be effective for pain management and are well-liked by users [38], however these don’t provide the Fabry MDT with as much disease-specific context. It must be noted that there is currently no evidence to indicate the efficacy of this approach in Fabry disease, and the use of a wearable was raised as an alternative option, particularly for children. This also resonated in the discussion of understanding treatment responses to pain medication (see later).

### Stressors and triggers of neuropathic pain in Fabry disease

Painful episodes and pain intensity in Fabry disease can be evoked by a range of stimuli, including physical touching or mechanical pressure, as well as both hot and cold temperature intolerance [4, 8, 13, 39]. These forms of evoked pain often manifest as stabbing, burning, tingling or shooting pain [4]. More-debilitating types of pain reported by patients are pain crises that are acute in nature, can last for extended periods and are triggered by heat, exercise stress or concurrent illness [8]. Severe gastrointestinal pain, including chronic pain between meals and intermittent pain after eating larger meal portions has been reported in Fabry disease [40].

Pain in Fabry disease can be reduced through awareness and avoidance of pain triggers or minimising activity using affected areas. The consensus across the panel was that this should be advised for all Fabry patients, and that they should be supported in this strategy, by the provision of clinical letters that helped them reduce pain triggers at school or in the workplace (Table 1).

1. Avoidance of stressors/triggers of neuropathic pain should be advised in all patients with Fabry disease.
2. Support for avoidance/triggers of neuropathic pain should be provided (e.g., clinical letters to schools or workplaces).

The experience of the Delphi panel was that many patients with Fabry disease could manage their pain in this way, and that most adopted such a strategy prior to presenting in clinic with painful symptoms. However, avoidance of triggers was seen as a strategy that may be effective in conjunction with analgesic medication, rather than as a pre-medication step, and the panel indicated that the needs of each patient should be managed in this context. Especially for children, the aim is to maximise their potential capacity for academic exposure and social interaction, and to develop a pain management strategy appropriate to this goal.

### First-line analgesic medication for neuropathic pain in Fabry disease

The management of pain in Fabry disease is an area that has lacked consensus. Pain can also be accompanied by significant anxiety and distress that requires mental-health support. Despite the availability of evidence-based recommendations for treatment of pain in Fabry disease [22, 24], there is not an established consensus for analgesic therapy in the UK.

In the first round Delphi survey, the most-commonly used first-line analgesic medications were the anticonvulsant medications carbamazepine, gabapentin and pregabalin. Amongst these, both carbamazepine and gabapentin were favoured over pregabalin in cases of Fabry pain, based on the experience of the panellists of efficacy and their evidence-based place in guidelines on neuropathic pain [25]. Pregabalin was prescribed where tolerance with carbamazepine or gabapentin was in question, although it is not recommended in paediatric subjects. When a consensus was sought, carbamazepine and gabapentin were considered as first-line analgesic treatments (Table 1).

Other medications that are used in the clinical setting for Fabry pain include tricyclic antidepressants (TCA) amitriptyline and nortriptyline, as well as the selective serotonin-noradrenaline re-uptake inhibitors (SSNRI) duloxetine and venlafaxine. Because TCAs and SSNRIs are antidepressant medications, they are used for treating Fabry patients with both neuropathic pain and low mood or clinical depression, rather than as first-line treatments for managing Fabry pain (Table 2). Since one of the consensus treatment goals (see above) is ‘to minimise psychological stress associated with neuropathic pain’, the use of TCAs and SSNRIs may be an option for treating Fabry pain in patients with reported or diagnosed anxiety and depression, however they are not considered first-line medications. The consensus was that:

1. Gabapentin can be used as a first-line analgesic medication for the treatment of neuropathic pain in Fabry disease.
2. Carbamazepine can be used as a first-line analgesic medication for the treatment of neuropathic pain in Fabry disease.

### Second-line analgesic medication for neuropathic pain in Fabry disease

In the first-round survey of Delphi panellists, a number of analgesic medications were reported as being used in cases of neuropathic pain in Fabry disease, including ibuprofen, tramadol, codeine, oxycodone and topical lidocaine. However, there was no consensus on their use in episodes of Fabry pain (Table 2). Morphine was considered as an option for Fabry crises, to be used with caution and only under the careful guidance of a pain team, with careful assessment and management of side effects. Similarly, capsaicin and lidocaine were reported as second-line options for severe cases of neuropathic pain under the prescription and guidance of the pain team, with criteria-based access.

### Treatment responses to neuropathic pain medications in Fabry disease

The need to effectively monitor the treatment response to pain management medications was an area that achieved a clear consensus (Table 1). Again, the value of patient-reported outcomes using pain diaries and/or smartphone apps to monitor the frequency and severity of their pain was emphasised. Similarly, the consistent use of objective pain-assessment tools was strongly supported, such as the BPI. A caveat in this context was the unknown reinforcement effect of repeated assessment potentially worsening perceived pain. The inclusion of a pain expert as part of the Fabry MDT, as previously discussed, was also highlighted for cases where Fabry patients had pain that was refractory to first line and second line analgesic medication.

1. Treatment responses to neuropathic pain medication should be actively monitored using pain diaries and/or pain apps/wearables.
2. Treatment responses to neuropathic pain medication should be actively monitored using repeated application of pain-assessment questionnaires.
3. For neuropathic pain that is refractory to first- and second-line medications, expert pain prescribers should be consulted.

### Managing painful gastrointestinal symptoms of Fabry disease

Patients with Fabry disease report painful gastrointestinal symptoms, typically the earliest manifestations of disease, including symptoms of irritable bowel syndrome (IBS), diarrhoea, abdominal pain, decreased appetite, cramps, nausea and vomiting [41], with an increased prevalence in females and children with Fabry disease [41].

In the first-round survey of the Delphi panellists, the reported prevalence of gastrointestinal pain was variable, and the most-frequently reported symptoms were abdominal pain and diarrhoea. Patients with Fabry disease were not routinely referred for investigation of gastrointestinal symptoms for an alternative diagnosis, other than as a consequence of their Fabry disease, and < 10% of patients received an alternative diagnosis.

In the second-round survey of the Delphi panellists, consensus was achieved only that (Table 1):

1. Metoclopramide can be used to treat symptoms of nausea/vomiting.
2. Loperamide can be used to treat symptoms of diarrhoea.

There was no consensus on using dietetic advice about meal constituents, portion sizes, meal timings and frequency to manage gastrointestinal symptoms and abdominal pain (Table 2), although interest was expressed in dietary interventions such as the low FODMAP^1^ diet that has shown efficacy in reducing pain in people with IBS [42, 43]. Similarly, there was no consensus that the anti-emetic domperidone can be used to treat symptoms of nausea/vomiting or gastrointestinal pain in patients with Fabry disease.

### Psychological support for patients with episodes of pain in Fabry disease

Pain in Fabry disease is associated with decreased quality of life [8, 44] and is a contributory factor in increased rates of low mood, anxiety and depression for patients with Fabry disease compared to the general population [45–48]. A clear association between pain, disease burden and depression in Fabry disease is emerging [48] which has previously been under-diagnosed [45]. The correlation between Fabry pain and poorer psychological or mental-health status could be seen as an obvious and inevitable consequence of the disease process. However, it must be emphasised that modifiable factors may be targeted as part of an active psychological intervention for people living with Fabry disease, with the aim of improving outcomes [45, 49]. This is reflected in the overall support across the panel that access to psychological services is an important part of pain management strategies for people with Fabry disease (Table 1). More specifically, consensus was achieved for the following:

1. Patients with Fabry disease pain should be assessed for associated symptoms of anxiety and/or depression using a validated scoring tool, for example, the Centre for Epidemiologic Studies Depression Scale (CES-D).
2. Selection of analgesic medication for neuropathic pain in Fabry disease should accommodate symptoms of anxiety and/or depression.
3. Cognitive behavioural therapy (CBT) should be considered for patients with Fabry disease pain and anxiety and/or depression.
4. Psychological services should be included in the MDT in cases of Fabry disease pain.

The recognition of pain as a contributory factor in Fabry-associated low mood, anxiety and depression was highlighted, in that consensus was not achieved that CBT should be considered for all patients with Fabry disease, independently of pain, even though non-pain variables are also identified as underlying decreased psychological functioning in cases of Fabry disease [48]. This underscores that Fabry disease is an extremely heterogenous condition and not everyone has pain or overt symptomology. Similarly, the consensus on inclusion of expert psychological practitioners as part of the MDT indicates that the interaction of pain with low mood and depression in Fabry disease does require management by expert practitioners. Notably, the consensus that the selection of analgesic medication should accommodate symptoms of anxiety and/or depression does indicate that antidepressant mediations such as TCAs and SSNRIs are of value, although they did not achieve consensus as first-line medication for Fabry pain.

## Discussion

**Fig. 2:**
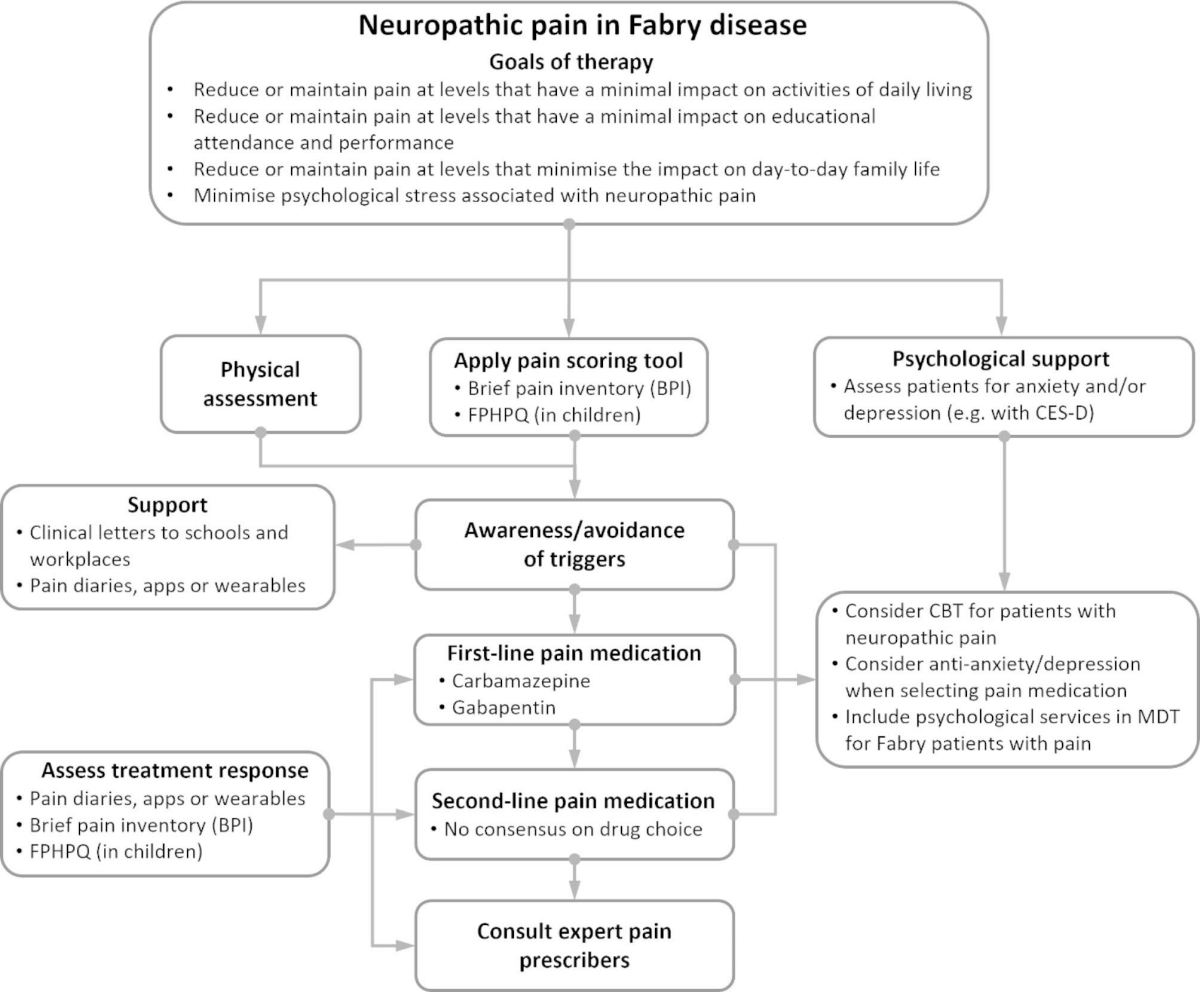
Algorithm for neuropathic pain based on the Delphi panel consensus on pain in Fabry disease. (The algorithm is a visual representation of the consensus of the Delphi panel. It illustrates the consensus outcomes as part of a process for management of neuropathic pain. It is intended to be a visual aid only, and not a formal guideline for pain management. BPI, Brief pain inventory; CBT, Cognitive behavioural therapy; CES-D Centre for Epidemiologic Studies Depression Scale; FPHPQ, Fabry-specific Pediatric Health and Pain Questionnaire; MDT, Multidisciplinary team)

Across the two consensus survey rounds, the Delphi panel responses indicate that different services apply different strategies for pain assessment and management in Fabry disease, based on their different experience and access to support services. However, the Delphi panel reached a consensus on 21 out of 41 aspects of pain assessment and management of pain in Fabry disease. These encompassed steps in the care pathway from the goals of therapy through to holistic support, and are summarised in a visual pathway in Fig. 2. Of note, was the consensus on assessment of pain in Fabry disease, which adhered to a straightforward approach, with use of the BPI as a tool for investigating the severity of pain in Fabry disease and its impact on functioning. Use of the BPI reflects its well-established validity for use in pain assessment across several clinical specialisms [36] and its recommended use for patients enrolled in Fabry-disease registries [37]. Consensus was also achieved for the training and use of Fabry-specific pain diaries or apps during assessment of pain in Fabry disease, since similar tools have demonstrated early efficacy and user engagement for non-Fabry chronic pain [38], although wearables may be more-suited to use in children, for whom the BPI is also less well-suited. Both the BPI and use of pain diaries are also suited to monitoring treatment responses to pain-management strategies in Fabry disease, and this was also a consensus amongst the panel.

The importance of understanding pain triggers was endorsed, along with the provision of support for patients with Fabry disease to manage their exposure to painful stressors and triggers in the school or workplace, for example with clinical letters that endorsed non-participation in activities that may result in painful episodes. The use of adjunct pain medication was endorsed at any point, in recognition that many people with Fabry disease will have self-managed exposure to pain triggers as much as possible prior to seeking clinical help.

The choice of first-line and second-line drug treatment revealed that a selection of pain medications was used for symptomatic pain control. Amongst these, carbamazepine or gabapentin were accepted as consensus first-line analgesic medication in cases of Fabry pain. The involvement of pain specialists as part of the MDT, and in making treatment recommendations, is consequently an important consensus criterion in managing pain in Fabry disease. The consistent use of pain assessment tools such as the BPI in monitoring treatment responses to pain medication is a consensus approach, as is the use of pain diaries during this phase of pain management.

Management of gastrointestinal pain revealed a consensus for management of gastrointestinal symptoms, such as nausea and vomiting, but in both survey rounds there was limited insight into strategies to address abdominal pain. This ultimately reflected the lack of clear options that emerged from the systematic literature review. Assessment and treatment of low mood, anxiety and depression generated a good consensus for formal assessment of these symptoms in patients experiencing Fabry pain, along with a consensus that analgesic strategies for pain should accommodate the antidepressant attributes of drugs used for pain management. A consensus on the value of considering CBT for patients with Fabry disease experiencing pain is clear, along with the inclusion of psychological expertise in the MDT for treating pain in Fabry disease.

### Strengths and weaknesses of the modified Delphi initiative

An accepted strength of the Delphi methodology is that it minimises the possibility of group bias centred on peer-pressure to agree with dominant opinions that can be a feature of face-to-face committee-style discussions. The anonymity of the responses provided by the Delphi panel members was maintained throughout the process. In a small community of rare-disease experts, the likely composition of the panel may have been predictable, but the attribution of individual responses to each round of survey questions was not disclosed at any point, including during the publication process. Similarly, the 100% response rate ensured the opinions expressed and consensus reached were a true reflection of the expert group. A limitation is that, although the 10 panel members represent a significant section of a small community of rare-disease specialists, the majority of this community were not part of the consensus exercise. This means that the generalisability of the consensus opinions cannot be guaranteed. A further limitation of this current Delphi method is that it did not further test the value of statements that did not meet the consensus threshold, even if there was a weight of opinion in favour of the stated outcome. An implicit limitation is that all panellists are metabolic disease specialists looking after patients with Fabry disease and not pain specialists in their own right. Lastly, this Delphi panel reflects the opinions and experience of expert healthcare professionals but did not include in its scope the experience and attitudes of people with Fabry disease.

## Conclusions and future directions

The modified Delphi initiative reported here achieved consensus on 21 aspects of the assessment and management of pain in patients with Fabry disease and should provide a foundation for establishing consistency in UK clinical practice. Despite the existence of guidelines for Fabry disease that include pain management [22, 24, 25] there is none with an established consensus and this issue is not specific to the UK. The consensus reported here should catalyse further discussion amongst stakeholders within the Fabry disease healthcare professional community, centred on developing specific consensus guidance for the management of pain in Fabry disease, particularly neuropathic pain. This provides an opportunity to develop pain ladders and analgesic medication choices that are particularly adapted to children and adults with Fabry disease. Patient-reported outcomes and the experience of acute and chronic pain for people living with Fabry disease will be important components of specific guidance in this context. Similarly, this Delphi panel has confirmed the need for defined clinical intervention and holistic support for people experiencing anxiety and depression as a consequence of living with pain as part of their Fabry disease. The consensus panel outcomes reported here are not intended to frame clinical rules for managing pain in Fabry disease but have highlighted strengths in current UK clinical practice, along with unmet needs on which to focus ongoing research and discussion. This consensus is intended to prompt the next steps towards developing clinical guidelines.

## Data Availability

Not applicable.

## Footnotes

1 Fermentable oligosaccharides, disaccharides, monosaccharides and polyols.
